# Meckel’s Cartilage in Mandibular Development and Dysmorphogenesis

**DOI:** 10.3389/fgene.2022.871927

**Published:** 2022-05-16

**Authors:** M. Kathleen Pitirri, Emily L. Durham, Natalie A. Romano, Jacob I. Santos, Abigail P. Coupe, Hao Zheng, Danny Z. Chen, Kazuhiko Kawasaki, Ethylin Wang Jabs, Joan T. Richtsmeier, Meng Wu, Susan M. Motch Perrine

**Affiliations:** ^1^ Department of Anthropology, The Pennsylvania State University, University Park, PA, United States; ^2^ Department of Computer Science and Engineering, University of Notre Dame, Notre Dame, IN, United States; ^3^ Department of Genetics and Genomic Sciences, Icahn School of Medicine at Mount Sinai, New York, NY, United States

**Keywords:** craniofacial development, skull, lower jaw, mandible, embryonic cartilage, embryonic bone, Crouzon syndrome, fibroblast growth factor

## Abstract

The *Fgfr2c* ^*C342Y/+*^ Crouzon syndrome mouse model carries a cysteine to tyrosine substitution at amino acid position 342 (Cys342Tyr; C342Y) in the fibroblast growth factor receptor 2 (*Fgfr2*) gene equivalent to a *FGFR2* mutation commonly associated with Crouzon and Pfeiffer syndromes in humans. The Fgfr2c C342Y mutation results in constitutive activation of the receptor and is associated with upregulation of osteogenic differentiation. *Fgfr2c^C342Y/+^* Crouzon syndrome mice show premature closure of the coronal suture and other craniofacial anomalies including malocclusion of teeth, most likely due to abnormal craniofacial form. Malformation of the mandible can precipitate a plethora of complications including disrupting development of the upper jaw and palate, impediment of the airway, and alteration of occlusion necessary for proper mastication. The current paradigm of mandibular development assumes that Meckel’s cartilage (MC) serves as a support or model for mandibular bone formation and as a template for the later forming mandible. If valid, this implies a functional relationship between MC and the forming mandible, so mandibular dysmorphogenesis might be discerned in MC affecting the relationship between MC and mandibular bone. Here we investigate the relationship of MC to mandible development from the early mineralization of the mandible (E13.5) through the initiation of MC degradation at E17.7 using *Fgfr2c* ^*C342Y/+*^ Crouzon syndrome embryos and their unaffected littermates (*Fgfr2c* ^*+/+*^). Differences between genotypes in both MC and mandibular bone are subtle, however MC of *Fgfr2c* ^*C342Y/+*^ embryos is generally longer relative to unaffected littermates at E15.5 with specific aspects remaining relatively large at E17.5. In contrast, mandibular bone is smaller overall in *Fgfr2c* ^*C342Y/+*^ embryos relative to their unaffected littermates at E15.5 with the posterior aspect remaining relatively small at E17.5. At a cellular level, differences are identified between genotypes early (E13.5) followed by reduced proliferation in MC (E15.5) and in the forming mandible (E17.5) in *Fgfr2c* ^*C342Y/+*^ embryos. Activation of the ERK pathways is reduced in the perichondrium of MC in *Fgfr2c* ^*C342Y/+*^ embryos and increased in bone related cells at E15.5. These data reveal that the Fgfr2c C342Y mutation differentially affects cells by type, location, and developmental age indicating a complex set of changes in the cells that make up the lower jaw.

## 1 Introduction

Crouzon syndrome, with an estimated birth prevalence of 16.5/1,000,000 (Cohen and Kreiborg, 1992) is possibly the mildest of *FGFR2* craniosynostosis syndromes (Azoury et al., 2017). In addition to premature closure of cranial sutures, Crouzon syndrome presents with facial anomalies (retrusion, beaked nose, ocular proptosis), absence of major hand and foot abnormalities (Johnson and Wilkie, 2011; Lu et al., 2020), and mandibular dysgenesis (Costaras-Volarich and Pruzansky, 1984; Boutros et al., 2007; Lu et al., 2019). Change in the spatial relationship of the mandible to the cranial base develops independently of mandibular shape deformity, and may influence functional capabilities including eating and speech (Lu et al., 2019). Additional dysmorphologies of the mandible and teeth remain poorly characterized for this condition, although it has been proposed significant mandibular morphological and growth abnormalities occur in children with Crouzon syndrome (Costaras-Volarich and Pruzansky, 1984; Boutros et al., 2007). Development of the lower jaw begins much earlier than these studies have been able to describe and includes growth of Meckel’s cartilage prior to mineralization of the mandible. We propose embryonic development of the lower jaw, including Meckel’s cartilage and mandibular bone, may be affected in Crouzon syndrome.

The FGFR2c C342Y mutation associated with Crouzon syndrome results in constitutive activation of the receptor and is most commonly associated with up-regulation of osteogenic differentiation (Yu et al., 2003; Perlyn et al., 2006; Heuzé et al., 2014). Mouse models of the FGFR2c C342Y mutation have been used to study the development of the Crouzon phenotype in multiple tissues of the head (Perlyn et al., 2006; Martínez-Abadías et al., 2013; Motch Perrine et al., 2017; Lee et al., 2018; Motch Perrine et al., 2019). However, this mutation can also affect other cell types including mesenchymal stem cells, (Baddoo et al., 2003; Miraoui et al., 2009; Martínez-Abadías et al., 2010; Maruyama et al., 2010; Deckelbaum et al., 2012; Yeh et al., 2012; Martínez-Abadías et al., 2013; Chen et al., 2014; Xu et al., 2017). FGFR activation induces major intracellular signaling pathways including RAS-MAPK (ERK1/2), PI3K-AKT, PLCγ-PKC, and signal transducer and activator of transcription (STAT) (Ornitz and Itoh, 2015). ERK pathway is active in the osteogenic progenitors and peripheral chondrocytes of MC (Parada et al., 2015) and is essential for the formation of the lower jaw. In *Wnt1-Cre;Erk2* ^*fl/fl*^ mice, NCC-specific conditional knockout of *Erk2* disrupts mandibular osteogenic differentiation resulting in micrognathia (Parada et al., 2015). In addition, conditional overexpression of *Fgfr2c* causes upregulation of phosphorylates ERK (p-ERK), resulting in craniofacial hypoplasia including reduction of the mandible in murine models (Lee et al., 2018). Precise regulation of ERK pathway appears critical for normal mandibular development, but its involvement in development of the lower jaw of *Fgfr2c* ^*C342Y/+*^ Crouzon mice has not been elucidated.

Meckel’s cartilage develops from the most cranial of the transient pharyngeal arches that comprise a series of repeated outgrowths on the lateral sides of the embryonic head. The most cranial, or first pharyngeal arch (PA1) organizes into two separate compartments in jawed vertebrates: the maxillary process cranially and the mandibular arch caudally. NCC from the mandibular arch condense and then differentiate into chondrocytes to form Meckel’s cartilage (MC), a rod-like structure providing shape to the lower jaw. The same NCC that form MC give rise to the dermal bones of the upper and lower jaws and at least part of two of the three middle ear bones (malleus, incus). The segmental arrangement and structural organization of the pharyngeal arches is highly conserved throughout evolution, though the number of PAs varies (Graham and Richardson, 2012; Shone et al., 2016), suggesting conservation of the molecular mechanisms and signals that govern their development and their contribution to the pharyngeal skeleton (Frisdal and Trainor, 2014).

**FIGURE 1:**
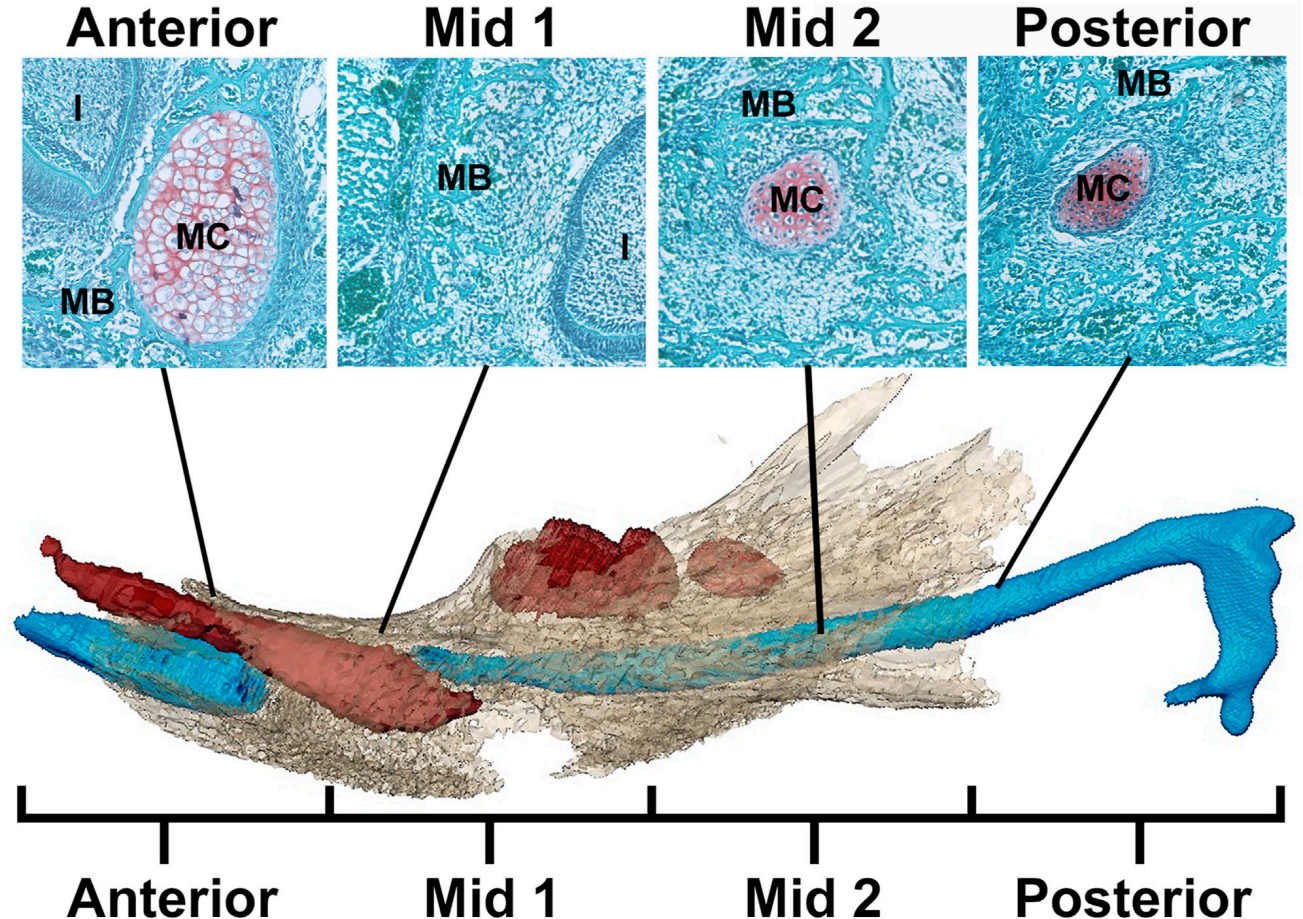
Schematic of MC in mandible identifying regions for analysis. Example histological sections (top) associated with developing mandible and MC (bottom) (after Svandova et al., 2020). In the histological sections, mandibular bone matrix (MB) is stained green and is adjacent to the red-orange MC stained using a standard Safranin-O protocol. Below, the developing mandible (tan), segmented from microCT images, is superimposed with MC (blue) and mandibular teeth (red) segmented from PTA-enhanced microCT images of an E17.5 *Fgfr2c* ^*+/+*^ embryo. Note that the color of MC and MB is not consistent between histological images (top) and 3D models created from PT-enhanced microCT images (bottom). MC is divided into 4 equal regions from anterior to posterior with Anterior and Mid 1 containing the break in MC at E17.5 associated with the root of the incisor (I). The incus is not included in this model.

The current paradigm of mandibular development assumes that Meckel’s cartilage (MC) functions as a model during early mandibular development and as a template for growth of the mandible later in development (Svandova et al., 2020). During development, the distal portion of MC becomes the symphysis, a point of fusion of the left and right hemi-mandibles, and the proximal portion of MC forms cartilages of the malleus and incus, bones that ossify endochondrally (McKenzie, 1958; Amin and Tucker, 2006; Frisdal and Trainor, 2014; Svandova et al., 2020). Mineralization of the mandibular bone begins at E14 laterally along MC and eventually encases it (Ramaesh and Bard, 2003). Part of the Mid and/or Posterior regions of MC (Figure 1) transforms into ligament, but other portions of the Mid region degrade and eventually completely disappear (Ishizeki et al., 2001; Svandova et al., 2020; Nakamura et al., 2021).

The scientific premise that MC and mandibular development are tightly coordinated implies that the relationship between MC and the forming mandible is critical and that dysmorphogenesis of either (or both) structure(s) can lead to anatomical abnormalities (Ishizeki et al., 1999; Manocha et al., 2019; Svandova et al., 2020). Typically, cells within each structure behave predictably and in an apparently coordinated fashion to create a fully functional lower jaw. Insults, including variants of the FGF/FGFR signaling pathway, can cause significant changes to cells in MC and the developing mandible that may affect the form and function of the mandible. Here, we use *Fgfr2c* ^*C342Y/+*^ Crouzon syndrome mice (Eswarakumar et al., 2004) that have characteristically shortened faces and commonly exhibit aberrant occlusion of the jaws. These mice are known to have abnormal osteogenesis and chondrocyte proliferation, as well as mandibular dysmorphology at birth (Eswarakumar et al., 2004; Holmes et al., 2018; Motch Perrine et al., 2019) and provide an appropriate model for investigating the direct effects of the FGFR2c C342Y mutation on MC and mandibular development. We hypothesize that this mutation will drive alterations in cell activity and structure within MC and the developing mandible resulting in aberrant mandibular form in embryonic *Fgfr2c* ^*C342Y/+*^ mice.

## 2 Materials and Methods

### 2.1 Mouse Model

The *Fgfr2c* ^*C342Y/+*^ mice were a generous gift from Dr. Eswarakumar, who previously described the generation of this model (Eswarakumar et al., 2004). The model was maintained on a CD1 background for viability and breeding. Embryos were collected at embryonic days E13.5, 15.5, and 17.5. PCR for sex and genotype was performed using a tail snipped at collection. We do not anticipate any differences due to sexual dimorphism based on our previous work on multiple mouse models (Motch Perrine et al., 2017; Motch Perrine et al., 2019; Lesciotto et al., 2022), still we have indicated the balance of males and females used in this study in Table 1. Mouse litters were produced in compliance with animal welfare guidelines approved by Icahn School of Medicine at Mount Sinai and Pennsylvania State University Animal Care and Use Committees (ISMMS #07-0757 and PSU #46558). To more precisely define developmental age, E13.5 and E15.5 embryos were staged using the Embryonic Mouse Ontogenetic Staging System, EMOSS (Musy et al., 2018). E17.5 embryos have developed beyond the capabilities of this system so are considered to be an average of 17.5 days post conception. Average developmental age at E13.5 was 341.14 h for *Fgfr2c* ^*C342Y/+*^ embryos and 338.4 h for *Fgfr2c* ^*+/+*^ embryos. At E15.5, average developmental age was 371.93 h for *Fgfr2c* ^*C342Y/+*^ embryos and 372.11 h for *Fgfr2c* ^*+/+*^ embryos.

**TABLE 1:** Specimen demographics.

	E13.5				E15.5				E17.5			
	*Fgfr2c* ^*C342Y/+*^		*Fgfr2c* ^*+/+*^		*Fgfr2c* ^*C342Y/+*^		*Fgfr2c* ^*+/+*^		*Fgfr2c* ^*C342Y/+*^		*Fgfr2c* ^*+/+*^	
	♂	♀	♂	♀	♂	♀	♂	♀	♂	♀	♂	♀
PTA microCT	3	2	3	2	2	3	3	3	3	1	4	1
microCT					2	3	3	3	3	1	4	1
Histology	1	2	2	1	2	1	1	2	2	1	1	2
Immunofluorescence					1	2	1	2				

### 2.2 Imaging Protocols

Phosphotungstic acid (PTA)-enhanced Microcomputed Tomography (microCT) images for MC and microCT images for bone analyses were acquired by the Center for Quantitative Imaging at the Pennsylvania State University (https://iee.psu.edu/labs/center-quantitative-imaging) using the General Electric v|tom|x L300 nano/microCT system. Five *Fgfr2c* ^*C342Y/+*^ and 5 *Fgfr2c* ^*+/+*^ embryos were scanned at E13.5, 5 *Fgfr2c* ^*C342Y/+*^ and 6 *Fgfr2c* ^*+/+*^ embryos were scanned at E15.5, and 4 *Fgfr2c* ^*C342Y/+*^ and 5 *Fgfr2c* ^*+/+*^ embryos were scanned at E17.5. Image data were reconstructed on a 2,024 × 2,024 pixel grid as a 32-bit volume, and were reduced to 16-bit volume for image analysis using Avizo 2019.3 and 2020.2 (Thermo Fisher Scientific). Scanning parameters varied from 60–100 kV and 75–170 μA, to accommodate age group and type of scan performed. Voxel sizes ranged from 6.9 to 15 microns (µm) for bone scans and 4.5–8 µm for PTA-enhanced scans.

### 2.3 Segmentation and Quantification of Cartilage

**FIGURE 2:**
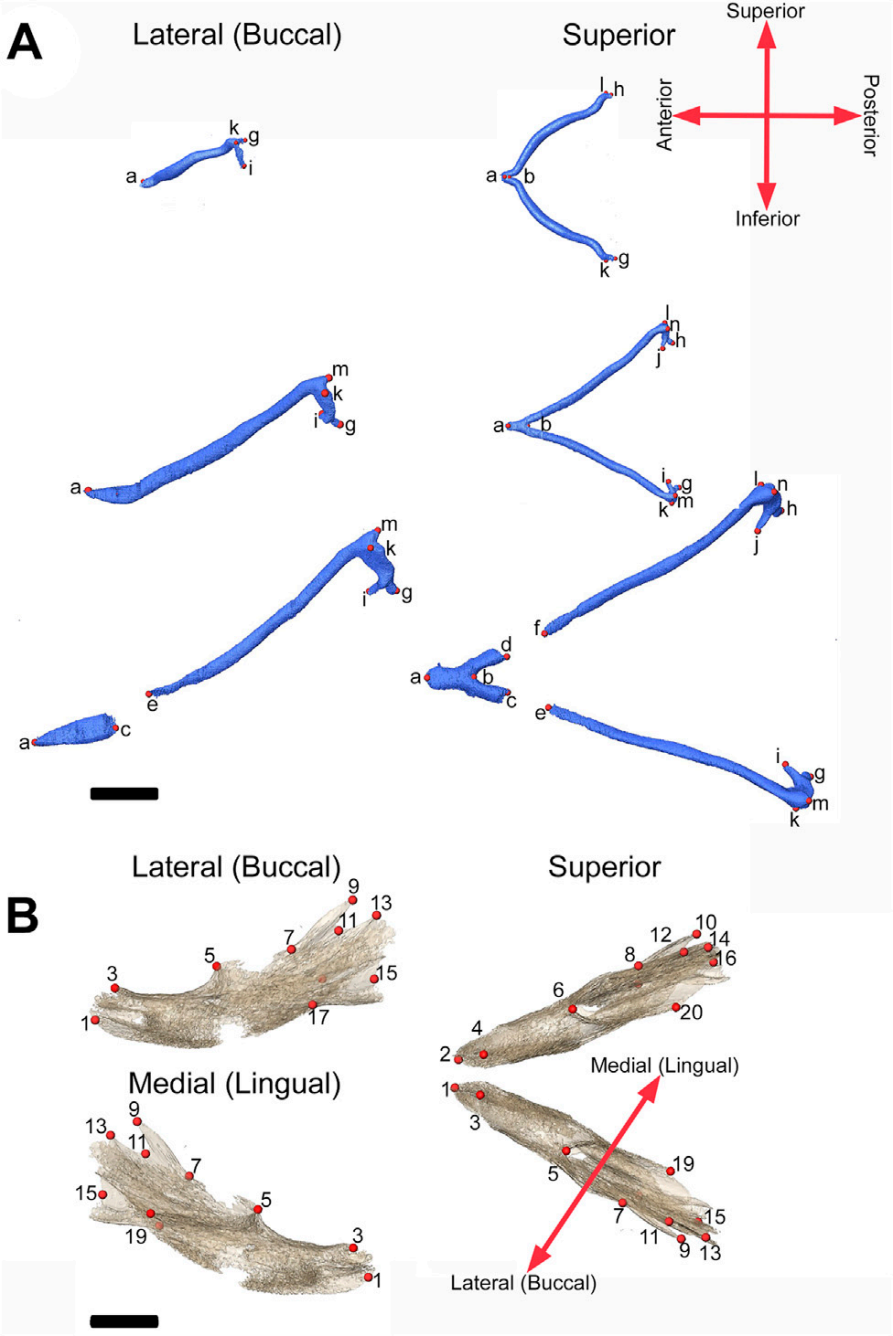
3D landmarks on developing MC and mandible. **(A)** Two medial landmarks and 8, 10, or 14 bilateral landmarks shown on MC at E13.5 (top), E15.5 (middle) and E17.5 (bottom) embryos respectively. Lateral views (buccal) are displayed on the left and superior views are on the right. **(B)** Ten bilateral landmarks are shown on the left hemi-mandible of an E17.5 embryo. Lateral views are displayed on the left (buccal view (top) and lingual view (bottom)) and superior view of the left and right hemi-mandibles are shown on the right. Scale = 1.0 mm.

3D models of MC were created from PTA-enhanced microCT (Lesciotto et al., 2020) images of specimens at E13.5, E15.5 and E17.5 (Table 1) using a previously reported automatic segmentation approach (Zheng et al., 2020). Due to the complex 3D cartilage structures and variations in our images as well as large volumes of image data, an effective and efficient automatic method is needed for our cartilage segmentation task. Recently, deep learning methods have achieved great successes in biomedical image segmentation (Ronneberger et al., 2015). Compared with common CT images, each of our 3D microCT volumes is very large (about 1,500 × 2,000 × 2,000 voxels per image), and consequently, it will be extremely difficult to rely on manual annotation to produce a sufficiently large amount of labeled data for training a deep learning segmentation model. Hence, our segmentation approach must be able to accommodate a large image size and make the most out of sparsely labeled training data. First, we chose a very sparse subset of 2D image slices (e.g., 2%–10%) in our 3D microCT training volumes for manual annotation that represents and covers the rest (unannotated) 2D slices of the training volumes well. Then, sparse manual annotations were conducted by experts (MKP and SMMP) using Avizo 2020.2 (Thermo Fisher Scientific). Next, the annotated 2D slices were used to train a judiciously designed fully convolutional network (FCN) model to identify target objects (called pseudo-labels or PLs) in all the unannotated 2D slices of the training volumes. In this way, we sought to bridge the large gap between our sparse annotation and full annotation of the entire 3D training volumes. Since our FCN model is not trained with a standard full annotation protocol (due to the sparse annotation used), its identified PLs on the unannotated 2D slices may be unreliable and noisy. Thus, we estimated the reliability of the PLs by computing the associated uncertainty maps of the PLs, which quantify the pixel-wise prediction confidence. With the expanded training set (i.e., 3D images with both manual labels and pseudo labels) and guided by the uncertainty maps, the FCN model was trained iteratively to distill more stable knowledge about the training data, thus becoming more robust and generalizing better to unseen data. Finally, the segmentation results were integrated along the three orthogonal planes (orthogonal to each of the *x, y*, and *z* directions) to further boost the overall segmentation accuracy (by average ensemble of segmentation results along the three directions). Results of automated segmentation were reviewed by an expert anatomist and segmentation was corrected manually if necessary. Volume and area were estimated using the Material Statistics of Avizo 2020.2 (ThermoFisher Scientific). Volume and area were compared between the genotypes within age categories with independent-samples Mann Whitney *U* test performed in IBM SPSS Statistics v 27 (IBM Corp). Once created, the 3D models of MC were quantified by collecting the 3D coordinates of landmarks designed for age-specific analyses (Figure 2; Table 2). Landmark collection as well as volume and area calculations were performed in Avizo 2019.3 and 2020.2 (Thermo Fisher Scientific). Each sample was digitized twice by the same observer, checked and corrected for gross error, and remaining measurement error was minimized by averaging the coordinates of the two trials. A maximum of 5% intraobserver error in landmark placement was accepted. Morphological differences were assessed as described in Section 2.5. PTA-enhanced microCT images were used to hand segment tooth germs visualized in Figure 1.

**TABLE 2:** Definitions of MC landmarks.

Landmarks (left, right)	Definition^a^	^a^E13.5	E15.5	E17.5
A	Anterior-most point on the tip of Meckel’s cartilage	x	x	x
B	Posterior-most point on the symphysis of Meckel’s cartilage	x	x	x
c, d	Posterior-most point on the portion anterior to the break in Meckel’s cartilage			x
e, f	Anterior-most point on the portion posterior to the break in Meckel’s cartilage			x
g, h	Posterior-most point on the arm of Meckel’s cartilage (processus brevis mallei)	x	x	x
i, j	Anteroinferior-most point on the arm of Meckel’s cartilage (manubrium mallei)	x	x	x
k, l	Lateral-most point on the posterior aspect of the arm of Meckel’s cartilage (caputo mallei)	x	x	x
m, n	Superior-most point on the posterior aspect of the arm of Meckel’s cartilage (caputo mallei, head of malleus)		x	x

a Definitions refer to positions with mandible in standard anatomical position (jaws in occlusion).

### 2.4 Segmentation and Quantification of Bone

Isosurfaces of mandibular bone were created by automated segmentation of bone from 16-bit microCT images using a minimum threshold of 70–100 mg/cm^3^ partial density hydroxyapatite based on phantoms scanned with the samples using Avizo 2020.2 (ThermoFisher Scientific). Data were processed using a median filter in Avizo 2020.2 (ThermoFisher Scientific) to remove noise and reviewed by an expert anatomist. No manual corrections to form were necessary for MB. Volume and area were estimated for each specimen using the Material Statistics module of Avizo 2020.2 (Thermo Fisher Scientific). Volume and area were compared between the genotypes within age categories with independent-samples Mann Whitney *U* test performed in IBM SPSS Statistics v 27 (IBM Corp). These isosurfaces were used to assess mandibular form by recording the 3D coordinates of 14 (7 bilateral) landmarks at E15.5 and 20 (10 bilateral) landmarks at E17.5 (Figure 2; Table 3) in Avizo 2019.3 (Thermo Fisher Scientific). Each sample was digitized twice by the same observer, checked and corrected for gross error, and measurement error was minimized by averaging the coordinates of the two trials. A maximum of 5% intraobserver error in landmark placement was accepted. Morphological differences were assessed as described in Section 2.5. Sample sizes for each genotype and embryonic age are listed in Table 1.

**TABLE 3:** Definitions of mandible bone landmarks.

Landmarks (left, right)	Definition^a^	E15.5	E17.5
1, 2	Inferior-most point on incisor alveolar rim at midline of the incisor	x	x
3, 4	Superior-most point on incisor alveolar rim	x	x
5, 6	Anterior-lateral edge of alveolar process where first molar intersects the alveolus (anterior molar alveolus)	x	x
7, 8	Intersection of base of coronoid process and molar alveolar rim (posterior molar alveolus)		x
9, 10	Apex of coronoid process, most posterior point		x
11, 12	Most anterior point along the rim connecting the coronoid and condylar processes		x
13, 14	Most posterior point on the cranial angle of the condyloid process	x	x
15, 16	Midpoint on the supero-inferior axis of the most posterior aspect of the angular process	x	x
17, 18	Anterior-most point of the angular process at its intersection with the body of the mandible on the inferior aspect	x	x
19, 20	Most medial (lingual) edge of the posterior-most aspect of the molar alveolar process	x	x

a Definitions refer to positions with mandible in standard anatomical position (jaws in occlusion).

### 2.5 3D Morphometric Analysis of Meckel’s Cartilage and Mandible

To statistically determine differences in MC and mandibular form between genotypes, we used Euclidian Distance Matrix Analysis (EDMA) (Lele and Richtsmeier, 1995; Lele and Richtsmeier, 2001). EDMA converts 3D landmark data into a matrix of all possible linear distances between unique landmark pairs and tests for statistical significance of differences between shapes using a boot-strapped hypothesis testing procedure. Differences of specific linear distances are statistically evaluated by a 90% confidence interval produced through a non-parametric bootstrapping procedure (Lele and Richtsmeier, 1995). Rejection of the null hypothesis of similarity for linear distances enables localization of differences to specific dimensions. EDMA analyses were performed using WinEDMA (Cole, 2002).

### 2.6 Superimposition of PTA-Enhanced and Bone Targeting MicroCT Images

Scans targeting soft tissues such as MC (PTA-enhanced microCTs) and scans targeting hard tissues such as MB were performed on each specimen as described in Section 2.2. MB is very difficult to fully segment from PTA-enhanced microCTs, so therefore both scan types were used. For figures with superimposition of MB and MC, isosurfaces of MC and MB were created as described in Sections 2.3,2.4, respectively. Landmarks 1, 2, and 15 (Table 3) were taken on MB in both scans in Avizo 2020.2 (ThermoFisher Scientific) for each individual for which a superimposition was required. A rigid transformation, which moves the points of the first set as close as possible onto the points of the second set (the sum of the squared distances between corresponding points is minimized) was computed from these landmarks for aligning the model (bone microCT scan) to the reference (PTA-enhanced microCT scan) in Avizo 2020.2 (ThermoFisher Scientific). The superimposition was adjusted slightly by hand if necessary following landmark transformation of images. Superimpositions were used only for figure creation. Analysis of individual tissues was performed on individual scans targeting the tissue of interest.

### 2.7 Histology

Specimen were collected (*n* = 3 per age per genotype) and fixed in 4% paraformaldehyde for 24 h. Care was taken to balance between sexes, and E17.5 specimens were decalcified in 0.25 M EDTA at pH 7.4 for 12 days. Isolated heads were processed for paraffin-based histology per established protocol (Behringer et al., 2013; Motch Perrine et al., 2021). Using a rotary microtome, 7 µm serial, coronal sections were cut and mounted on Superfrost-Plus slides (Thermo Fisher Scientific), and the range of slides containing Meckel’s cartilage was determined for each specimen. Slide ranges were then divided into four equal regions (Anterior, Mid 1, Mid 2, Posterior, Figure 1), and one slide was selected per region per specimen for each stain. For immunohistochemistry, slides were subjected to epitope retrieval using an autoclave (121°C, 10 min) and sodium citrate buffer pH 6.0 before blocking with 3% hydrogen peroxide and 1% goat serum/bovine serum albumin. Sections were incubated with primary antibodies at room temperature for 2 h, Proliferating Cell Nuclear Antigen (PCNA, AbCam, ab18197, 1:2,000) or for 1 h, alkaline phosphatase (ALP, ab108377, 1:500). After washing in PBS, slides were incubated with HRP conjugated secondary antibody for one hour at room temperature (ab6721, 1:500) and Diaminobenzidine (DAB, Vector Laboratories) chromagen was used to identify immunoreactive structures (Durham et al., 2019). Safranin-O and tartrate-resistant acid phosphatase (TRAP) staining were performed per established protocol (Howie et al., 2018). Slides were imaged using a Leica BX50 microscope, DFC450 camera, and LAS-X imaging software (Leica Biosystems). Regions of interest (MC, mandible) were identified and analyzed using ImageJ color deconvolution and masks to count stained areas by color (Supplementary Figure S1) (Ruifrok and Johnston, 2001; Haub and Meckel, 2015; Durham et al., 2018; Landini et al., 2021). Careful gating for cell size (3–30 µm) was used to reduce noise in the form of stained areas smaller or larger than a cell. The same gating parameters were applied to every image analyzed. At least two images were captured per slide for each stain for each specimen investigated. Osteoclasts were identified by TRAP positivity and multiple nuclei and were counted by a single investigator (ELD) two times with significant correlation between counts. Counts were averaged for investigation. Non-parametric Mann-Whitney *U* tests were used where necessary to compare genotypes at each age, and then by each region using SPSS 25 software (IBM).

### 2.8 Immunofluorescence

Specimens (*n* = 3 per genotype) collected at E15.5 were fixed in 4% paraformaldehyde overnight, embedded in OCT compound, and frozen. Samples were sectioned at 10 µm of thickness on the cryostat. For immunofluorescence, sections were permeabilized in PBST (0.2% Triton X 100 in PBS) for 5 min. Antigen retrieval was performed at 100°C for 10 min in citrate buffer (Sigma-Aldrich, C9999). Sections were then blocked with 3% bovine serum albumin in PBS. Anti-p-ERK1/2 (Cell Signaling Technology, 4370) was diluted at 1:100 with PBS and incubated on the sections for 1 h. After washing with PBS, secondary antibody (1:500, Thermofisher, A32732) was incubated for 1 h. The sections were rinsed briefly in PBS and stained with 0.5 μg/ml 4′,6-Diamidino-2-Phenylindole (DAPI; Thermo Fisher Scientific) in PBS before mounting in anti-fade mounting medium (Vector Labs, H-1700). Slides were imaged using a Nikon Eclipse E 600 fluorescence microscope. Images were analyzed using ImageJ.

## 3 Results

### 3.1 Orientation of Meckel’s Cartilage and Mandible During Development

**FIGURE 3:**
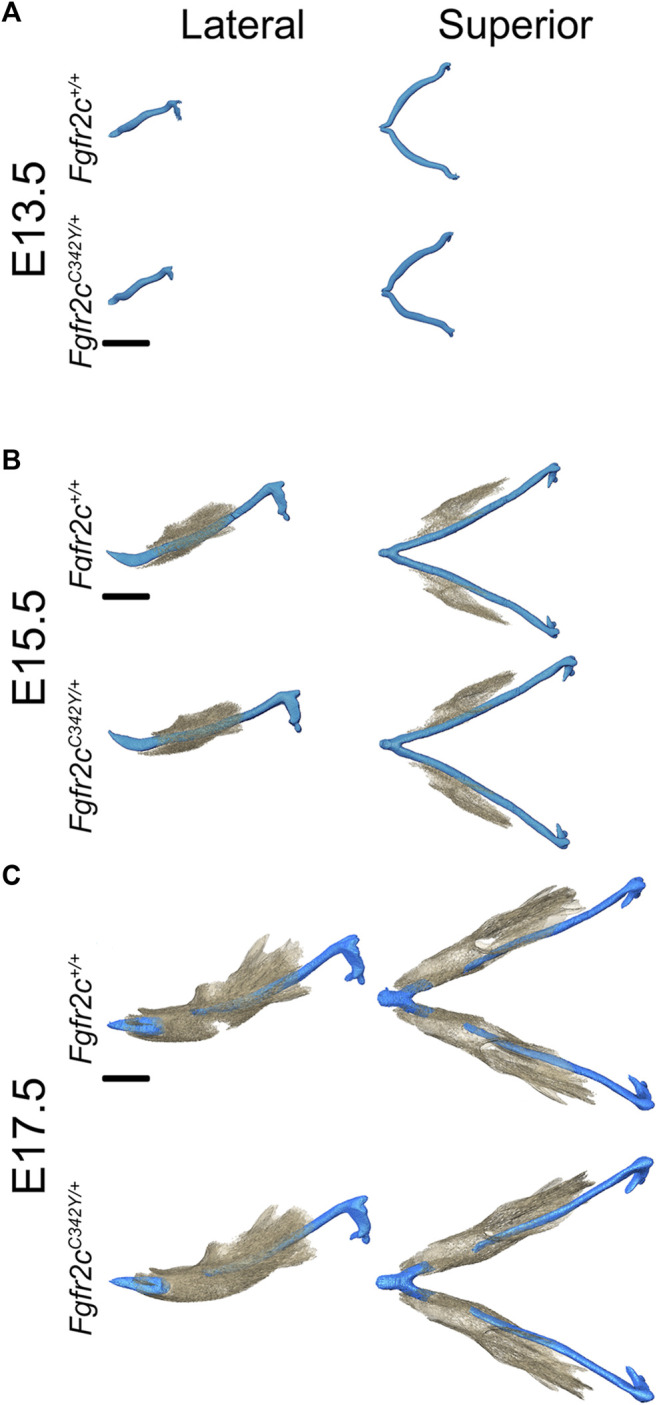
Relative orientation of MC and developing mandible in *Fgfr2c* ^*+/+*^ and *Fgfr2c* ^*C342Y/+*^ embryos. Segmentations of multimodal images of MC (blue) and mandibular bone (tan) of developing mandible at E13.5 **(A)**, E15.5 **(B)**, and E17.5 **(C)** with representative *Fgfr2c* ^*+/+*^ embryos displayed in the top row and *Fgfr2c* ^*C342Y/+*^ Crouzon syndrome embryos displayed in the bottom row of each age group. Lateral views (buccal) are displayed on the left and superior views on the right. Not enough bone is mineralized at E13.5 to be detected by microCT. Scale = 1.0 mm.

Visual inspection of superimpositions of bone and cartilage segmentations indicated only subtle morphological differences between *Fgfr2c* ^*C342Y/+*^ and *Fgfr2c* ^*+/+*^ embryos from E13.5 through E17.5 (Figure 3). Very thin areas of bone with little mineralization may not be detected at the indicated bone threshold in microCT images, as compared to traditional histological staining techniques.

### 3.2 Impact of Fgfr2c C342Y Mutation on Meckel’s Cartilage

**FIGURE 4:**
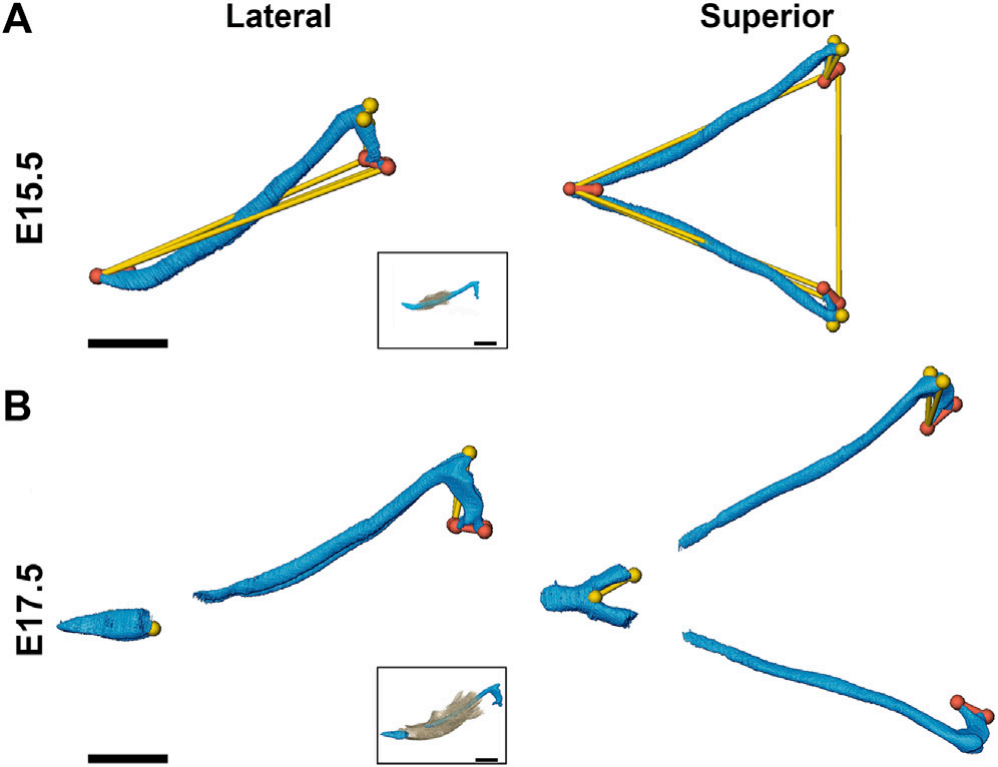
Morphological differences of MC in *Fgfr2c* ^*C342Y/+*^ embryos and *Fgfr2c* ^*+/+*^ littermates at E15.5 and E17.5. Linear distances that are statistically significantly different between genotypes by confidence interval testing (*α* = 0.10) are shown for E15.5 **(A)** and E17.5 **(B)**. Thin yellow lines indicate linear distances that are between 5% and 10% larger in *Fgfr2c* ^*C342Y/+*^ embryos as compared to *Fgfr2c* ^*+/+*^ embryos and thicker orange lines indicate linear distances that are between 10% and 20% larger in *Fgfr2c* ^*C342Y/+*^ embryos as compared to *Fgfr2c* ^*+/+*^ embryos. For orientation, superimpositions of MC and mandibular bone are inset. Lateral views (buccal) are displayed on the left and superior views are on the right. Scale = 1.0 mm.

At E13.5, *Fgfr2c* ^*C342Y/+*^ and *Fgfr2c* ^*+/+*^ embryos do not exhibit significant differences in MC form. However, at E15.5 MC volume is significantly larger in these embryos as compared to *Fgfr2c* ^*+/+*^ littermates (Table 4) and MC is significantly longer in the anteroposterior dimension (Figure 4A). MC of *Fgfr2c* ^*C342Y/+*^ embryos is not larger overall at E17.5 but linear distances characterizing the most anterior and most posterior aspects of MC are larger relative to unaffected littermates (Figure 4B).

**TABLE 4:** Results of Mann-Whitney tests and descriptive statistics for cartilage and bone volume by age and genotype.

	Mean		SD		*p*-Value
	*Fgfr2c* ^*C342Y/+*^	*Fgfr2c* ^*+/+*^	*Fgfr2c* ^*C342Y/+*^	*Fgfr2c* ^*+/+*^	
MC					
E13.5	0.06750	0.07679	0.00525	0.00884	0.095
E15.5	0.31588	0.27779	0.01747	0.01371	**0.008** ^a^
E17.5	0.36942	0.31859	0.01442	0.04183	0.066
Mandible					
E15.5 (Right)	0.1426	0.1163	0.02893	0.02757	0.177
E15.5 (Left)	0.1410	0.1231	0.02779	0.03771	0.429
E17.5 (Right)	0.7191	0.5987	0.21580	0.12847	0.171
E17.5 (Left)	0.7141	0.5982	0.22453	0.13215	0.257

a Statistically significant differences (*p* ≤ 0.05) highlighted with bold text.

**FIGURE 5:**
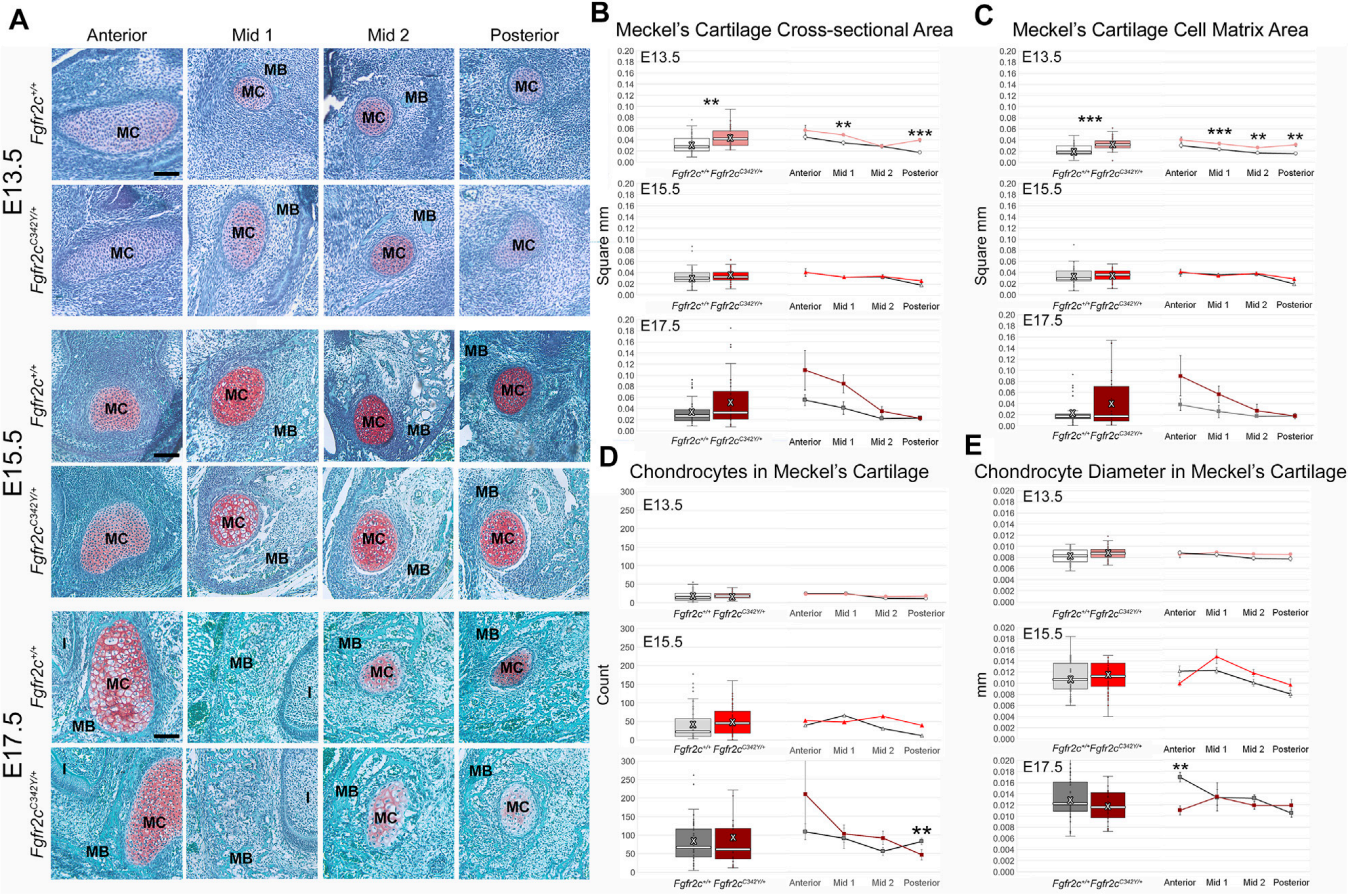
Histomorphometric analysis of MC in *Fgfr2c* ^*C342Y/+*^ embryos and *Fgfr2c* ^*+/+*^ littermates, E13.5, E15.5, E17.5. **(A)** Representative Safranin O-stained sections of MC and surrounding mandible bone (MB) in the coronal plane from *Fgfr2c* ^*+/+*^ and *Fgfr2c* ^*C342Y/+*^ embryos at E13.5 (top two rows), E15.5 (middle two rows) and E17.5 (bottom two rows). Chondrocytes and matrix are identified by the red-orange Safranin O stain with green bone matrix surrounding (MB). The incisor (I) is adjacent to MC and MB in Anterior and Mid 1. Scale = 0.10 mm. **(B)** Assessment of cross-sectional area of MC indicates some differences by genotype early in development (E13.5, top) with *Fgfr2c* ^*C342Y/+*^ embryos having a relatively greater area overall (box plot, left) and at specific regions in the antero-posterior dimension (line plots, right). **(C)** Similarly, more cartilage matrix was identified at E13.5 (top) in *Fgfr2c* ^*C342Y/+*^ embryos with no differences by genotype being noted later in development [E15.5 (middle), E17.5 (bottom)]. **(D)** The number of chondrocytes within MC did not vary by genotype at either E13.5 (top) or E15.5 (middle), but fewer chondrocytes were counted in Posterior of *Fgfr2c* ^*C342Y/+*^ embryos at E17.5 (bottom). **(E)** The average diameter of chondrocytes varied little from anterior to posterior aspects of MC and not at all by genotype at E13.5 (top) and E15.5 (middle). Chondrocytes in Anterior of E17.5 *Fgfr2c* ^*C342Y/+*^ embryos were smaller than those in *Fgfr2c* ^*+/+*^ embryos (bottom two rows). White bar in box plot identifies median. X in box plot identifies mean. *n* = 3 individuals per genotype per age with at least 4 images per region per embryo being analyzed. **p ≤ 0.01, ***p ≤ 0.001.

Effects of the Fgfr2c C342Y mutation on chondrocytes within MC are not qualitatively apparent, especially at the earliest time points investigated here (Figure 5A). Since asymmetry was not noted in our assessment of MC form, we did not separate left and right in our histological assessment. The area of MC measured in coronal sections of E13.5 embryos revealed increased area of MC in *Fgfr2c* ^*C342Y/+*^ Crouzon syndrome embryos (*p* = 0.001) as compared to *Fgfr2c* ^*+/+*^ littermates. This was driven by significantly increased area in Mid 1 (*p* = 0.003) and Posterior (*p* ≤ 0.001) regions of MC in *Fgfr2c* ^*C342Y/+*^ embryos at E13.5. No statistically significant differences in cross-sectional area were identified at the later timepoints (E15.5, E17.5) (Figure 5B). Since cartilage growth can result from increased matrix deposition (Figure 5C), net increase in cells (Figure 5D), or an increase in chondrocyte hypertrophy (cell diameter) (Figure 5E), we investigated these characteristics in MC and identified more matrix area in *Fgfr2c* ^*C342Y/+*^ embryos at E13.5 (*p* ≤ 0.001) relative to *Fgfr2c* ^*+/+*^littermates. This was the result of greater matrix in all regions in *Fgfr2c* ^*C342Y/+*^ embryos, but Mid 1 (*p* = 0.003), Mid 2 (*p* = 0.001), and Posterior (*p* = 0.001) regions had statistically significantly more cartilage matrix as compared to *Fgfr2c* ^*+/+*^ littermates (Figure 5C). Interestingly, there were no differences in chondrocyte number between genotypes at E13.5 or E15.5. The number of chondrocytes within MC was statistically significantly decreased in Posterior of *Fgfr2c* ^*C342Y/+*^ embryos at E17.5 (Figure 5D). Our assessment of the diameter of chondrocytes within MC indicated no differences by genotype at E13.5 and E15.5, however there were significantly smaller cells in Anterior of MC in *Fgfr2c* ^*C342Y/+*^ embryos at E17.5 (Figure 5E). This result correlates with the trend toward more cells in *Fgfr2c* ^*C342Y/+*^ embryos in Anterior at E17.5 (Figure 5D).

### 3.3 *Fgfr2c* Mutation Affects Proliferation in and Around Meckel’s Cartilage

**FIGURE 6:**
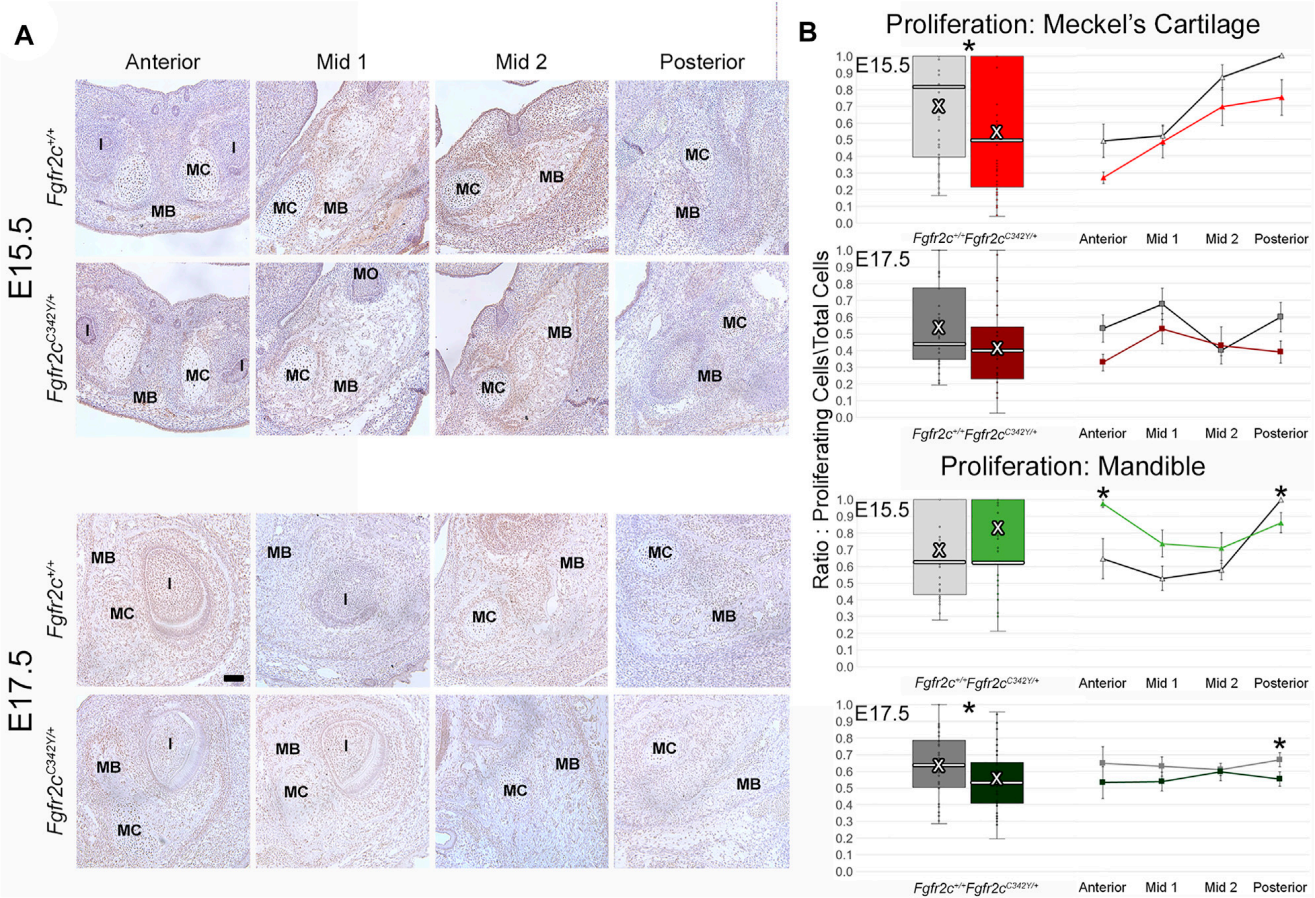
Effect of Fgfr2c C342Y mutation on proliferation in and around MC in E15.5 and E17.5 embryos. **(A)** Representative coronal sections of MC and surrounding mandible bone (MB) in *Fgfr2c* ^*+/+*^ and *Fgfr2c* ^*C342Y/+*^ embryos stained with Proliferating Cell Nuclear Antigen (PCNA, brown stain) counterstained with hematoxylin (purple) at E15.5 (top rows) and E17.5 (bottom rows). Scale = 0.1 mm. Note that due to variation in the location of the natural break in MC, only a small remnant of MC remains in Mid 1 at E17.5 in the displayed image of an *Fgfr2c* ^*+/+*^ embryo. **(B)** Quantification of proliferating cells in MC (top 2 rows) and mandible (bottom 2 rows) standardized by the total number of cells within each region. Proliferation was reduced in MC of *Fgfr2c* ^*C342Y/+*^ embryos at E15.5 and in mandible of *Fgfr2c* ^*C342Y/+*^ embryos at E17.5. White bar in box plot identifies median. X in box plot identifies mean. *n* = 3 individuals per age per genotype with at least 4 images per region per embryo being analyzed. *p ≤ 0.05. I = incisor; MO = molar.

Because we identified some significant differences in cell number between genotypes, we investigated proliferation to see if the mutation influenced chondrocyte and/or bone lineage cells during initial ossification. E15.5 and E17.5 embryos were used because the bone was robust enough at these ages for analysis. Differences in proliferation in MC are subtle (Figure 6A; Supplementary Figure S1), however an overall decrease in proliferation in chondrocytes from *Fgfr2c* ^*C342Y/+*^ embryos was identified at E15.5 (Figure 6B, top) relative to *Fgfr2c* ^*+/+*^ littermates. This relationship was no longer significant by E17.5 (Figure 6B, row 2). Within the forming mandible adjacent to MC, proliferation was increased in Anterior, and decreased in Posterior of E15.5 *Fgfr2c* ^*C342Y/+*^ embryos relative to *Fgfr2c* ^*+/+*^ littermates (Figure 6B, row 3). At E17.5 an overall decrease in proliferation in the mandibles of *Fgfr2c* ^*C342Y/+*^ embryos was driven by significantly less proliferation in Posterior (Figure 6B, bottom).

### 3.4 Impact of *Fgfr2c* Mutation on Mandible

**FIGURE 7:**
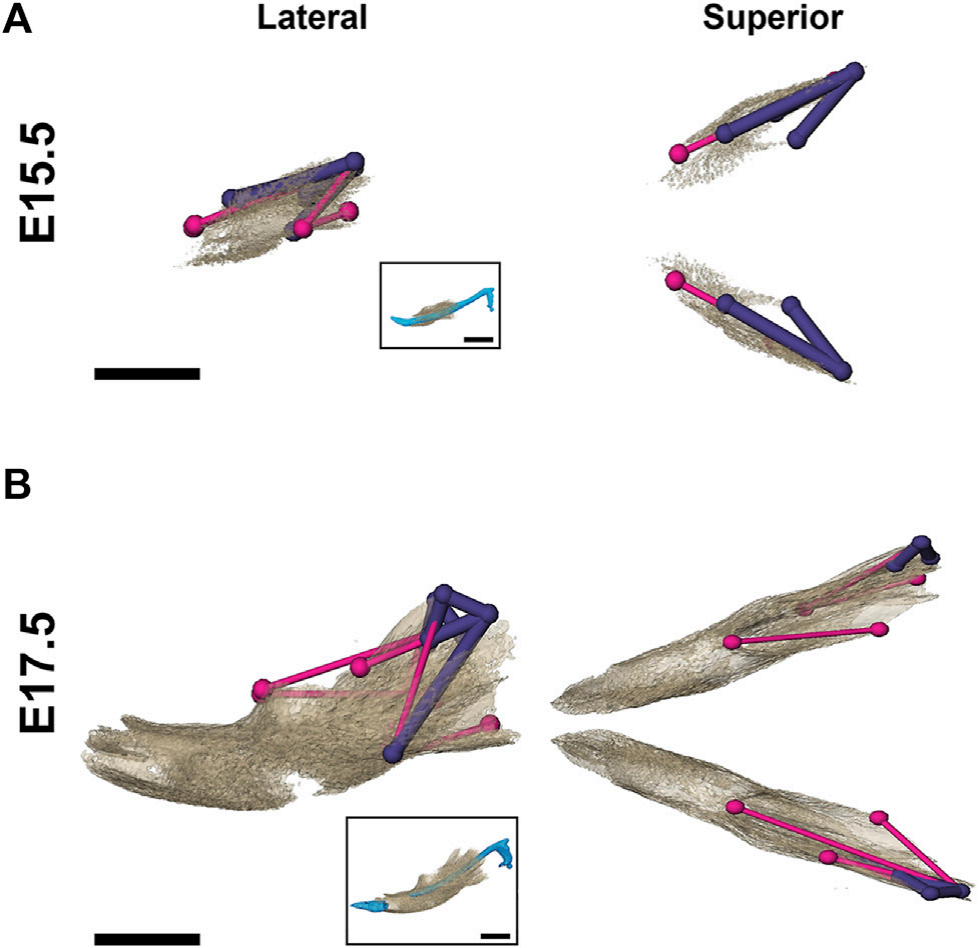
Morphological differences in *Fgfr2c* ^*C342Y/+*^ and *Fgfr2c* ^*+/+*^ mandibular bone at E15.5 and E17.5. Results of EDMA using 3D landmark coordinates of 14 anatomical landmarks at E15.5 **(A)** and 20 anatomical landmarks at E17.5 **(B)** show linear distances within each model that are significantly smaller by at least 5% between genotypes. Lateral views (buccal) at left and superior views at right. Thin pink lines indicate linear distances that are decreased by 5%–10% in *Fgfr2c* ^*C342Y/+*^ embryos, and thick purple lines indicate linear distances that are reduced by 10%–23% in *Fgfr2c* ^*C342Y/+*^ embryos. For orientation, superimpositions of MC and mandibular bone are inset. Scalebar = 1.0 mm.

As demonstrated by EDMA of 14 anatomical landmarks (Figure 2B; Table 3), the mandible is generally smaller in the antero-posterior dimension of *Fgfr2c* ^*C342Y/+*^ embryos relative to *Fgfr2c* ^*+/+*^ littermates at E15.5 (Figure 7A). EDMA of 20 anatomical landmarks (Figure 2B; Table 3) indicated differences between genotypes are concentrated in the posterior of the developing mandible corresponding to an area that stretches from the posterior portion of Mid 1 through the rostral portion of Posterior of MC that includes the coronoid, condylar, and angular processes (Figure 7B). There was no overall difference in bone volume at E15.5 or E17.5 (Table 4).

**FIGURE 8:**
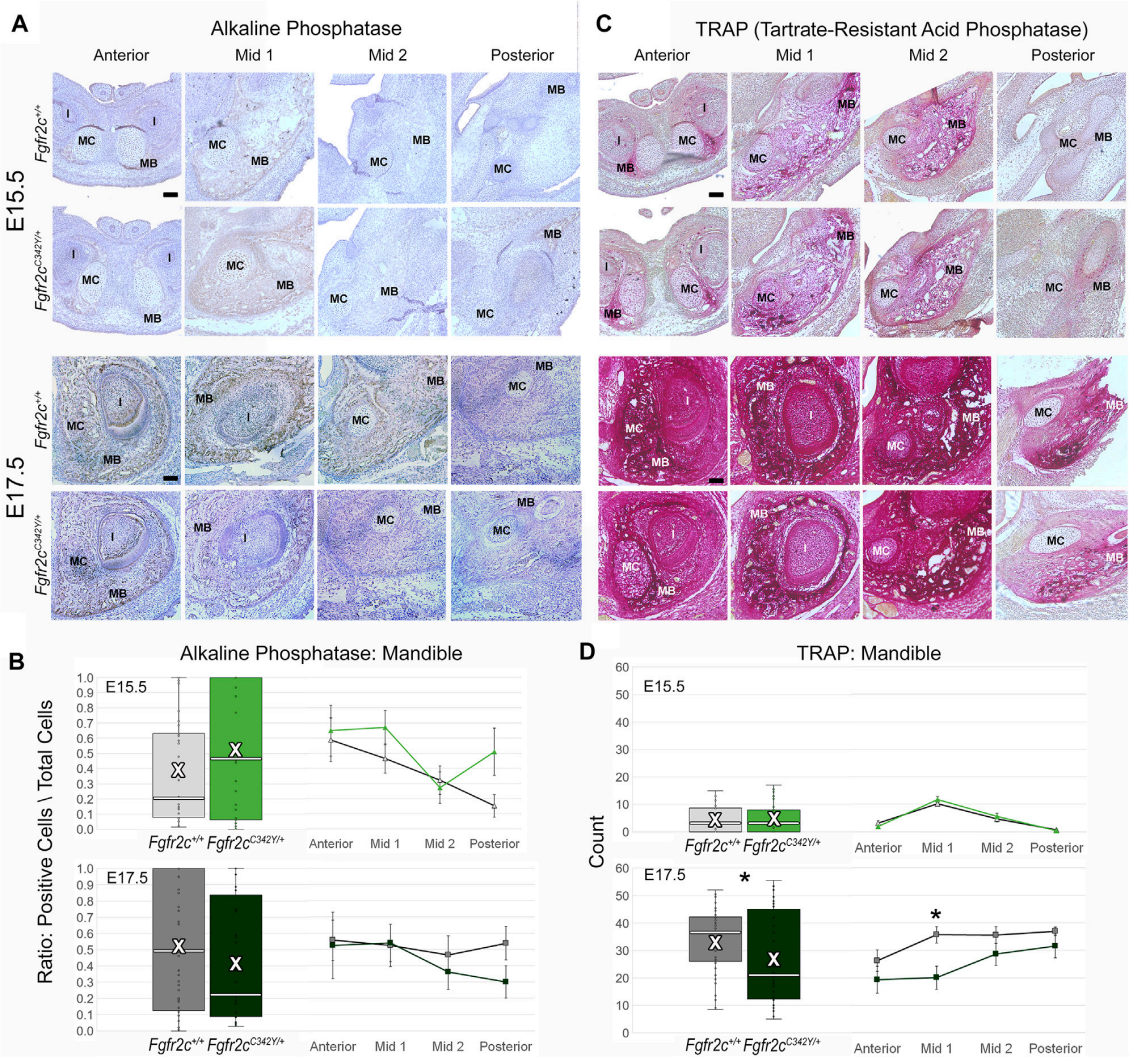
Immunohiostochemical analysis of osteoblasts and osteoclasts in developing mandible of *Fgfr2c* ^*C342Y/+*^ embryos and *Fgfr2c* ^*+/+*^ littermates. **(A,C)** Representative coronal sections of MC and surrounding mandible in *Fgfr2c* ^*+/+*^ (top rows) and *Fgfr2c* ^*C342Y/+*^ (bottom rows) embryos stained with alkaline phosphatase (ALP, **(A)** and Tartrate-Resistant Acid Phosphatase (TRAP, **(C)** at E15.5 (top) and E17.5 (bottom). Reduced TRAP staining can be noted in *Fgfr2c* ^*C342Y/+*^ images especially at Mid 1. **(B)** Quantification of ALP positive osteoblasts in the developing mandible standardized by the total number of cells within bone matrix. **(D)** Count of TRAP positive multi-nucleate osteoclasts in the mandible bone matrix. White bar in box plot identifies median. X in box plot identifies mean. *n* = 3 individuals per age per genotype with at least 4 images per region per embryo being analyzed. * = *p* ≤ 0.05.

To assess differentiation of osteoblasts, which are responsible for morphological changes in the mandible of *Fgfr2c* ^*C342Y/+*^ embryos, we investigated the levels of ALP-positive osteoblasts, which are responsible for bone mineralization (Hall, 2014), but identified no significant effects of this mutation on the ratio of alkaline phosphatase positive osteoblasts to total cells in the mandible (Figures 8A,B). In contrast to the lack of effect on the number of bone building osteoblasts, bone resorbing osteoclasts (measured by TRAP staining and multi-nucleation) were reduced in *Fgfr2c* ^*C342Y/+*^ embryos at E17.5 relative to *Fgfr2c* ^*+/+*^ littermates. This overall reduction in osteoclasts was driven by a significant reduction in osteoclasts in Mid 1 of *Fgfr2c* ^*C342Y/+*^ embryos (Figures 8 C,D).

### 3.5 Differential Effects of Fgfr2c C342Y Mutation on ERK Pathway Activation in Meckel’s Cartilage and Mandible

**FIGURE 9:**
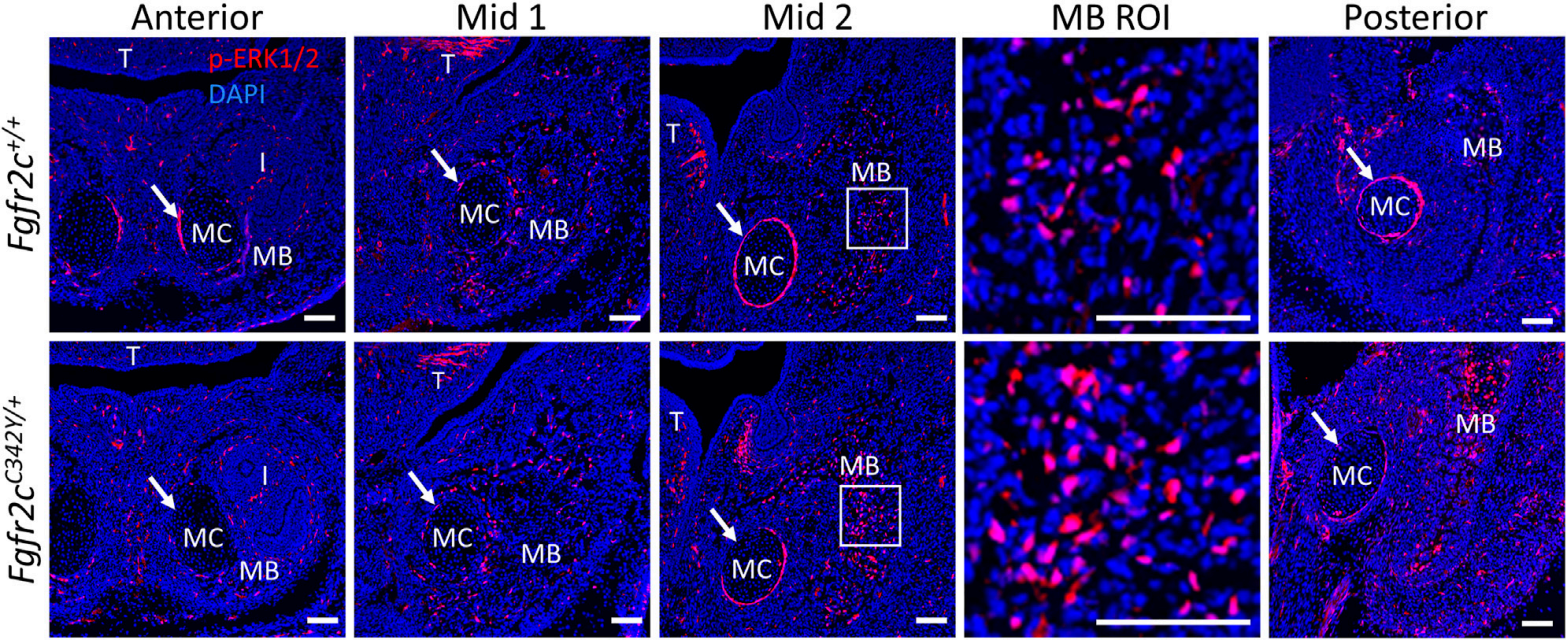
ERK pathway activation in the lower jaw of *Fgfr2c* ^*+/+*^ and *Fgfr2c* ^*C342Y/+*^ embryos at E15.5. Immunofluorescence for p-ERK1/2 in coronal sections of *Fgfr2c* ^*+/+*^ (top) and *Fgfr2c* ^*C342Y/+*^ (bottom) embryos at E15.5. Anterior, Mid 1, Mid 2, and Posterior indicate the regions of the lower jaw identified in Figure 1. Images in MB ROI column show higher magnification of the boxed areas in Mid 2. Arrows mark the locations of the perichondrium of MC. I, incisor; MB, mandible; MC, Meckel’s cartilage; T, tongue. *n* = 3 per genotype with at least 4 images per individual being analyzed. Scale = 100 µm.

To assess how Fgfr2c C342Y alters the ERK pathway in the lower jaw, we analyzed the expression of p-ERK1/2 in MC and mandible at E15.5, the earliest timepoint when the phenotypic effects of Fgfr2c C342Y mutation on MC and mandible were observed, prior to significant degradation in MC (Sakakura et al., 2007). p-ERK1/2 was detected in the mandible, tongue, tooth, peripheral chondrocytes in MC and perichondrium of MC by immunostaining (Figure 9). Little phosphorylated ERK was identified within MC, however there was significant activation in the perichondrium particularly in Anterior, Mid 2, and Posterior of MC, though this activation was reduced in E15.5 *Fgfr2c* ^*C342Y/+*^ embryos as compared to *Fgfr2c* ^*+/+*^ littermates. For mandibular bone, the expression level and pattern of p-ERK1/2 in Anterior and Mid 1 of *Fgfr2c* ^*C342Y/+*^ embryos were similar to *Fgfr2c* ^*+/+*^ littermates, however, ERK activation is increased in Mid 2 and Posterior. Our assessment of the downstream effects of the Fgfr2c mutation indicates that the ERK pathway is activated particularly in the posterior portion of the mandible of *Fgfr2c* ^*C342Y/+*^ embryos at E15.5 (Figure 9), matching the area of greatest morphological differences at that age (Figure 7).

## 4 Discussion

Though the morphological differences between MC and the developing mandible of *Fgfr2c* ^*C342Y/+*^ and *Fgfr2c* ^*+/+*^ embryos are subtle, our data demonstrate that this mutation has differential significant effects by tissue type and time at the cellular and morphological levels. We identified a larger, aberrantly formed MC in *Fgfr2c* ^*C342Y/+*^ embryos after E14 when mineralization of the mandible has already begun (Ramaesh and Bard, 2003). However, our data reveal a relatively small mandible in *Fgfr2c* ^*C342Y/+*^ embryos at E15.5 and E17.5. Similarly, investigations of newborn and postnatal *Fgfr2c* ^*C342Y/+*^ mandibles found them to be relatively small (Khominsky et al., 2018; Motch Perrine et al., 2019). These observations suggest a larger MC gives way to a smaller mandible during the latter days of prenatal development.

Overall, the significant differences in MC between genotypes identified by histomorphometry were intermittent in time and patchy in anatomic distribution. Our findings of increased cross-sectional area and matrix area in *Fgfr2c* ^*C342Y/+*^ E13.5 embryos were not associated with any significant changes in MC morphology at E13.5, but *Fgfr2c* ^*C342Y/+*^ MC becomes relatively larger later in development. These data suggest that even subtle changes early in development can produce significant morphological effects later, or that there are additional/alternate cellular processes at work.

Observations of growth plate cartilages in long bones identify chondrocyte proliferation, hypertrophy, and matrix deposition as the cellular processes that contribute to cartilage growth (Breur et al., 1991; Wilsman et al., 2008; Cooper et al., 2013). No differences between genotypes in MC area, contribution of matrix, chondrocyte number, or chondrocyte size were identified at E15.5, and proliferation was reduced in *Fgfr2c* ^*C342Y/+*^ embryos at E15.5. Few morphological and cellular differences between genotypes were identified at E17.5 as well. Though it is possible that statistically insignificant changes in cell size, cell number, and contribution of matrix can have biologically significant effects on development through additive and/or complementary mechanisms, it is also possible that MC increases in size using processes other than those identified in growth plate cartilages (Kaucka et al., 2017). Further, compensatory or redundant growth mechanisms for cartilage and bone might also explain why we did not identify dramatic effects of this mutation. The lack of consistent differences in MC morphology and cell behaviors between genotypes over time calls for alternate approaches focused on additional, potentially influential processes that produce affects that differ by cell type and developmental stage.

Investigation of the downstream targets of FGFR2 activation confirmed our assessment that the effects of the Fgfr2c C342Y mutation are cell type specific. ERK1/2 is the main effector of FGF signaling (Brewer et al., 2016; Ray et al., 2020). ERK pathway promotes osteoblast differentiation and bone formation *in vitro* and *in vivo* (Xiao et al., 2000; Xiao et al., 2002; Kim et al., 2019). In a previous study, *Fgfr2c* ^*C342Y/+*^ mice exhibited upregulated p-ERK in osteogenic fronts of the coronal suture compared with controls (Shibata et al., 2014; Lee et al., 2018). Our results show that ERK phosphorylation is increased in mandibular bone related cells and decreased in cartilage related perichondrium in the *Fgfr2c* ^*C342Y/+*^ embryos. From these assessments, we can conclude that the effects of constitutive activation of the FGF pathway are cell type specific.

The assertion that MC functions as a model for developing bony tissues of the mandible early in development and as a template for growth later in development is not corroborated by our analysis. Not only do the embryonic *Fgfr2c* ^*C342Y/+*^ mandible and MC differ in opposite ways relative to their unaffected littermates, but we see few consistent changes in cellular activity in the four lower jaw regions identified across the embryonic times assayed. Likewise, in mice in which the Sox9 gene, a transcription factor essential for endochondral bone formation, was conditionally inactivated in cranial neural crest cells, cartilage derived from these cells and endochondral bones were totally absent. However, the mandible still formed in these mice though it was small and misshapen (Mori-Akiyama et al., 2003). Though we know MC lengthens and increases in diameter with growth, the cell processes responsible for these and other morphological changes in MC have been studied by only a few investigators (e.g., Sakakura et al., 2007; Shibata et al., 2014; Shibata et al., 2014; Kaucka et al., 2017) and are not well defined.

Maintaining the balance between proliferation and differentiation is crucial for osteogenesis. Alkaline phosphatase, which identifies osteoprogenitors, pre-osteoblasts and differentiated osteoblasts (Huang et al., 2007) can be detected in the mandible as early as E11 (Shibata and Yokohama-Tamaki, 2008). Though no significant differences between genotypes were identified by alkaline phosphatase staining, reduced proliferation and increased ERK activity in the mandible indicate a shift from proliferation to osteoblast differentiation in the mandible, particularly posteriorly at E15.5 (Figure 9). Interestingly, this result correlated with the location of the most significant morphological differences consistent across the age groups revealed by EDMA (Figure 7). Our results suggest that subtle tissue-specific imbalance between proliferation and differentiation triggered by the Fgfr2c C342Y mutation may contribute to the small morphological changes of MC and mandible. In addition, the tissue, time, and region-specific effects of this mutation can impact the relationship between developing MC and mandible. Our results prompt the hypothesis that seemingly small effects combine additively or by interaction to subtly change MC and mandible morphology. Further, Modifying the relationship between these important structures may produce aberrant scaffolding that contributes to dysmorphogenesis associated with this mutation.

## Data Availability

The raw data supporting the conclusion of this article will be made available by the authors, without undue reservation. Bone microCT and PTA-enhanced microCT data used for this study are also available on Penn State University Libraries ScholarSphere; doi:10.26207/qgke-r185.
