# Supplementary figures and images for: Meckel’s Cartilage in Mandibular Development and Dysmorphogenesis
Source: Front Genet. 2022 May 16;13:871927. doi: 10.3389/fgene.2022.871927 (PMC9149363; doi:10.3389/fgene.2022.871927)

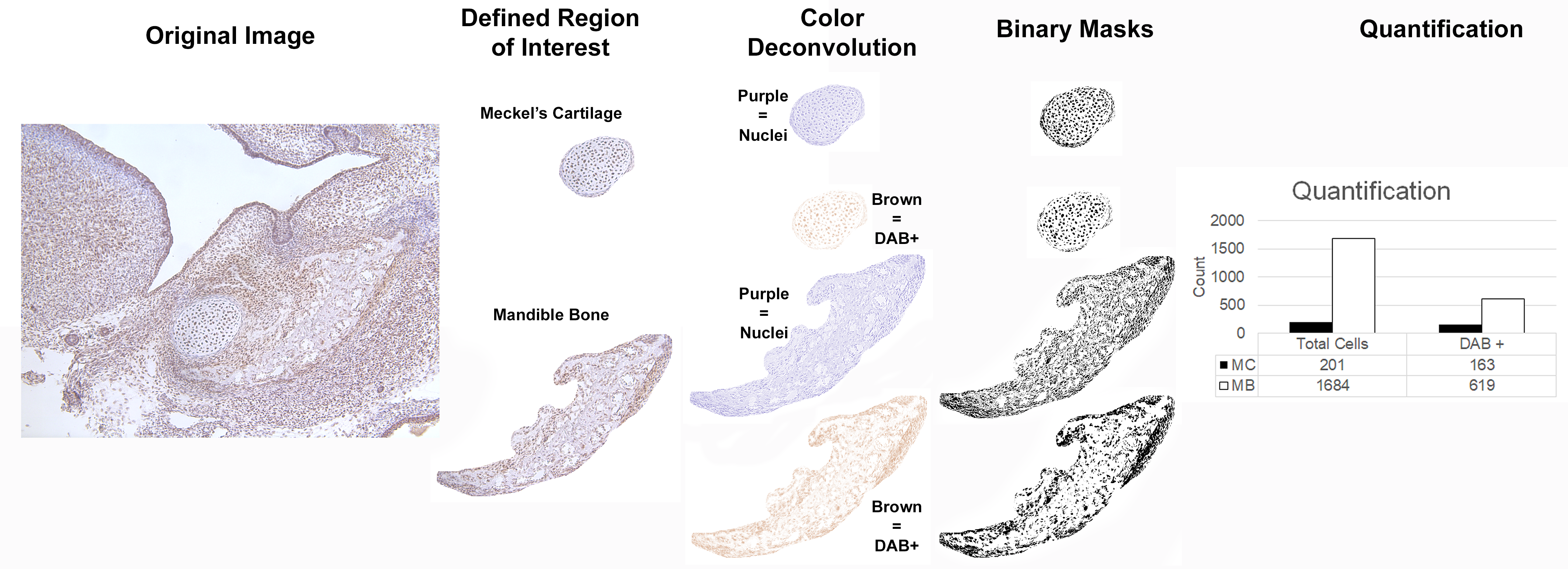

Supplement: Supplementary file 1 [file Image1.TIF]
